# Akut symptomatische epileptische Anfälle in Assoziation mit COVID-19

**DOI:** 10.1007/s10309-021-00443-y

**Published:** 2021-09-30

**Authors:** Matthias Mauritz, Eugen Trinka

**Affiliations:** 1 Universitätsklinik für Neurologie, Christian Doppler Klinik, Paracelsus Medizinische Universität und Centre for Cognitive Neuroscience, Ignaz-Harrer-Str. 79, 5020 Salzburg, Österreich; 2 Department of Public Health, Health Services Research and Health Technology Assessment, UMIT – University for Health Sciences, Medical Informatics and Technology, Hall in Tirol, Österreich; 3 Neuroscience Institute, Christian Doppler Klinik, Paracelsus Medizinische Universität und Centre for Cognitive Neuroscience, Salzburg, Österreich

**Keywords:** Pandemie, Epilepsie, Enzephalopathie, SARS-Cov-2, Pandemic, Epilepsy, Encephalopathy, SARS-CoV‑2

## Abstract

Akut symptomatische epileptische Anfälle treten in einem engen zeitlichen Zusammenhang zu einer akuten strukturellen oder funktionellen Schädigung des Gehirns auf, die viele verschiedene Ursachen haben kann. Neurologische Symptome wie Enzephalopathie, Geruchsstörung und Kopfschmerzen finden sich häufig bei COVID-19. Epileptische Anfälle ereignen sich nur in 1–2 % aller mit COVID-19 hospitalisierten Patient*innen. Eine mögliche direkte Invasion des ZNS durch SARS-CoV‑2 sowie indirekte Effekte durch Hypoxie, Inflammation und metabolische Entgleisungen werden als Ursache für die neurologischen Manifestationen bei COVID-19 angenommen.

## Abstract (en)

Acute symptomatic epileptic seizures occur in close temporal relationship with an acute structural or functional damage of the brain, which can have many different causes. Neurological symptoms, such as encephalopathy, anosmia and headache have been frequently reported in patients with COVID-19. Epileptic seizures occur in only 1–2% of all persons hospitalized with COVID-19. A possible direct effect by invasion of the CNS by SARS-CoV‑2 and indirect effects due to hypoxia, inflammation and metabolic derangements, have been discussed as potential causes of the neurological manifestations in COVID-19.

Im Dezember 2019 wurde in Wuhan, China, das Auftreten eines Clusters von Patient*innen mit interstitiellen Pneumonien unbekannter Ursache berichtet. Anfang Januar 2020 wurde als Pathogen ein neues Betacoronavirus identifiziert und aufgrund der phylogenetischen Ähnlichkeit mit dem Erreger des *Severe Acute Respiratory Syndrome* (SARS) als *Severe Acute Respiratory Syndrome Coronavirus 2* (SARS-CoV-2) bezeichnet [48]. Die von SARS-CoV‑2 ausgelöste Erkrankung wird Coronavirus Disease 2019 (COVID-19) genannt. Bislang sind Stand 16.05.2021 weltweit rund 162 Mio. Menschen an COVID-19 erkrankt und 3,3 Mio. Menschen an den direkten Folgen der Erkrankung verstorben [46].

## Neurotropismus von SARS-CoV-2

Bereits kurz nach dem Beginn der COVID-19-Pandemie wurden erste Berichte über das Auftreten von neurologischen Manifestationen im Rahmen von COVID-19 Erkrankungen veröffentlicht. Eine initiale retrospektive Studie von 214 mit bestätigter SARS-CoV-2-Infektion hospitalisierten Patient*innen aus Wuhan, China, beschrieb das Auftreten von neurologischen Symptomen in 36,4 % [32]. Einzelne Fallberichte von Enzephalitis in Assoziation mit SARS-CoV-2-Infektionen als mögliche Manifestation einer direkten ZNS-Invasion des Virus wurden veröffentlicht [5, 35, 38]. Der Nachweis von SARS-CoV-2-RNA mittels PCR aus dem Liquor gelang aber nur selten, und die klinische Evidenz für direkt durch SARS-CoV‑2 vermittelte Enzephalitiden ist bislang gering. Es lagen bislang aus Untersuchungen zu den saisonalen Coronaviren und SARS-CoV/MERS-CoV experimentelle und klinische Evidenz für das Potenzial von Coronaviren, das Nervensystem von Menschen zu befallen, vor [6]. Es existieren Fallberichte von Enzephalitis, akut disseminierter Enzephalomyelitis (ADEM), Schlaganfällen und verschiedenen neuromuskulären Manifestationen bei Patient*innen mit SARS und MERS [14]. Auch bei anderen respiratorischen Virusinfektionen wie Influenza wurden neurologische Komplikationen wie Enzephalopathie und Enzephalitis, epileptische Anfälle oder Guillain-Barré-Syndrome berichtet [36]. Es ist bekannt, dass der ACE2-Rezeptor, durch dessen Bindung SARS-CoV und SARS-CoV‑2 menschliche Zellen infizieren, an verschiedensten Stellen im ZNS, besonders auf Neuronen im Hirnstamm sowie auf Endothelzellen exprimiert wird [31]. Als mögliche Routen einer direkten Invasion von SARS-CoV‑2 in das ZNS wurden unter anderem die Infektion von olfaktorischen Neuronen im Riechepithel und der konsekutive retrograde axonale Transport des Virus in das ZNS, die Überwindung der Blut-Hirn-Schranke im Rahmen einer Virämie und/oder durch Infektion von Endothelzellen und der Transport des Virus in das ZNS durch infizierte Leukozyten postuliert [14, 37]. Bisherige Ergebnisse aus neuropathologischen Studien und Liquoruntersuchungen zu SARS-CoV‑2 stellen dessen Virulenz für das Nervensystem teilweise infrage: So konnte beispielsweise die RNA des Virus in den Zellen der Riechschleimhaut, nicht aber eindeutig in den olfaktorischen Neuronen nachgewiesen werden [20, 33]. Zudem wird der ACE2-Rezeptor von Zellen der Riechschleimhaut, nicht aber von olfaktorischen Neuronen exprimiert [7]. Mehrere Autopsieserien konnten SARS-CoV‑2 entweder nicht oder nur in geringen Mengen im Gehirn nachweisen [42]. Ein systematischer Review zu Liquoranalysen bei Patient*innen mit COVID-19 und neurologischen Symptomen fand positive SARS-CoV-2-PCR-Analysen in nur 17 von 304 (6 %) Fällen [29]. In manchen Fällen wurden hohe Cycle-threshold-Werte angegeben, sodass mögliche Kontaminationen vermutet wurden [20]. Derzeit werden als Ursache für die meisten neurologischen Manifestationen in Assoziation mit COVID-19 überwiegend indirekte Mechanismen und Auswirkungen der systemischen Erkrankung auf das Gehirn wie metabolische Entgleisungen (z. B. Hyponatriämie, Urämie), Hypoxie, Inflammation, Hyperkoagulopathie und endotheliale Dysfunktion angenommen [20].

## Neurologische Manifestationen von COVID-19

Neurologische Symptome und Manifestationen werden bei Patient*innen mit COVID-19 in verschiedenen Arbeiten in unterschiedlicher Häufigkeit, je nach Studie zwischen 3,5–84 %, gefunden [21, 47]. Die berichteten neurologischen Manifestationen umfassen Enzephalopathie, Geruchs- und Geschmacksstörung, Kopfschmerzen, zerebrovaskuläre Erkrankungen wie ischämischer Schlaganfall, intrazerebrale Blutungen und zerebrale Sinusvenenthrombosen, epileptische Anfälle, hypoxische Hirnschädigung sowie para-/postinfektiöse Syndrome wie Guillain-Barré-Syndrom, akute disseminierte Enzephalomyelitis (ADEM) und akute nekrotisierende Enzephalopathie [20]. Eine in mehreren Krankenhäusern in New York City über 2 Monate durchgeführte, prospektive Beobachtungsstudie von 4491 mit COVID-19 hospitalisierten Patient*innen fand ein Auftreten von neuen neurologischen Manifestationen in 14 % [17]. Die ersten neurologischen Symptome traten im Median 2 Tage nach den ersten COVID-19-Symptomen auf. Als häufigste neue neurologische Diagnose wurde eine metabolische Enzephalopathie festgestellt (6,8 %), gefolgt von Schlaganfällen (1,9 %), epileptischen Anfällen (1,6 %) und hypoxischer Enzephalopathie (1,4 %). Es wurden in dieser Arbeit keine Fälle von Meningitis, Enzephalitis oder Myelitis in Verbindung mit SARS-CoV‑2 identifiziert. Patient*innen, bei denen eine neue neurologische Manifestation auftrat, waren schwerer von COVID-19 betroffen, was sich z. B. an einer signifikant höheren Rate an invasiver Beatmung oder an einem längeren Spitalaufenthalt zeigte. Zudem hatten Patient*innen mit neuen neurologischen Erkrankungen in dieser Arbeit eine signifikant höhere Mortalität gegenüber Patient*innen mit COVID-19 ohne neurologische Manifestationen [17]. Neben den akuten neurologischen Komplikationen werden neurokognitive Beeinträchtigungen wie mentale Erschöpfung, Gedächtnisstörungen und dysexekutives Syndrom als Langzeitfolgen einer COVID-19-Erkrankung berichtet [34, 40].

## Akut symptomatische epileptische Anfälle in Assoziation mit COVID‑19

In der bislang größten Untersuchung zu neurologischen Manifestationen bei COVID-19 von Frontera et al. fanden sich epileptische Anfälle bei 74 von 4491 Patient*innen (1,6 %) und stellten damit nach Enzephalopathie und Schlaganfällen die dritthäufigste neurologische Manifestation dar. Bei 34 der 74 Patient*innen (46 %) war bislang keine Diagnose einer Epilepsieerkrankung bekannt gewesen [17]. In einer systematischen Übersichtsarbeit der Literatur zu neurologischen Manifestationen bei COVID-19 wurden epileptische Anfälle etwas seltener in 0,7 % der insgesamt 68.361 untersuchten Fälle angegeben [8]. Das Auftreten von epileptischen Anfällen bei Patient*innen mit COVID-19 wurde auch in mehreren Fallberichten und Fallserien beschrieben [3, 22].

Epileptische Anfälle können das Symptom einer COVID-19-Erkrankung sein, das zur Erstvorstellung der Patient*innen in einer Notaufnahme führt. In einer Untersuchung von allen über einen Zeitraum von 2 Wochen hospitalisierten COVID-19-Patient*innen im Iran, hatte in 45 von 5872 Fällen (0,8 %) ein epileptischer Anfall zur Aufnahme in das Krankenhaus geführt [24]. Nur 9 % dieser COVID-19-Patient*innen mit epileptischen Anfällen hatten eine Anamnese von Epilepsie. Ähnliche Ergebnisse fanden sich bei 1043 mit COVID-19 über einen Zeitraum von 6 Wochen hospitalisierten Patient*innen in Boston, USA. In dieser Kohorte war bei 7 Patient*innen (0,8 %) ein epileptischer Anfall das präsentierende Symptom bei Krankenhausaufnahme gewesen [2]. Davon hatten 3 Patient*innen keinerlei andere COVID-19-Symptome wie Husten oder Fieber vor dem Anfall gezeigt. Bei 3 Patient*innen war eine Epilepsieerkrankung bekannt gewesen, in den 4 anderen Fällen fand sich bei 2 Patient*innen ein Schlaganfall in der Anamnese. Bei 3 Patient*innen fanden sich schwere metabolische Entgleisungen in Form von Hyponatriämie, Urämie und dialysepflichtigem Nierenversagen.

Epileptische Anfälle treten bei 15–20 % aller kritisch kranken Patient*innen auf Intensivstationen auf. Die meisten dieser Anfälle zeigen keine klinisch erkennbaren Zeichen und können nur mittels Anwendung einer kontinuierlichen EEG-Ableitung („continuous EEG monitoring“ [cEEG]) detektiert werden [12]. Zwei unterschiedliche Arbeiten, bei denen bei jeweils 22 COVID-19-Patient*innen aufgrund von Enzephalopathie oder vorausgegangenen klinischen Anfallsereignissen eine cEEG-Ableitung durchgeführt wurde, fanden bei allen Patient*innen eine Verlangsamung der Hintergrundaktivität. Bei jeweils rund einem Drittel der Patientin*innen fanden sich epileptiforme Potenziale [18, 30]. Zwei Patient*innen mit elektroenzephalographischen Anfällen, die keine Anamnese einer Epilepsieerkrankung hatten und bei denen eine Bildgebung des Gehirns keine akute strukturelle zerebrale Pathologie gezeigt hatte, wurden berichtet. In einer weiteren cEEG-Studie von 111 COVID-19-Patient*innen, von denen sich rund drei Viertel zum Zeitpunkt der EEG-Ableitung auf der Intensivstation befanden und eine schwere Bewusstseinsstörung aufwiesen, fanden sich bei 30 % epileptiforme Potenziale und bei 7 % epileptische Anfälle [39]. Bei 4 % der Patient*innen wurden ausschließlich nichtkonvulsive bzw. elektroenzephalographische Anfälle beobachtet.

Status epilepticus (SE) bei Patient*innen mit COVID-19 wurde ebenfalls in Fallberichten beschrieben [1, 10, 45]. Ein systematischer Review zu SE und COVID-19 fand 47 Fälle eines SE in Assoziation mit einer SARS-CoV-2-Infektion [13]. Nur 3 Patient*innen in dieser Kohorte hatten eine bekannte Epilepsieerkrankung. Für die meisten SE-Fälle wurde eine akut symptomatische Ursache des SE angenommen, allerdings konnte die Ätiologie des SE in 55 % nicht identifiziert werden. In 14,9 % der Fälle wurde die Ätiologie des SE als „vaskulär“ (intrazerebrale Blutung, posteriores reversibles Enzephalopathiesyndrom [PRES], ischämischer Schlaganfall) beurteilt, gefolgt von „septisch“ in 10,6 % und „inflammatorisch“ in 8,6 %. Bei 4 Patient*innen mit SE wurde eine positive SARS-CoV-2-PCR aus dem Liquor berichtet. Bei der Mehrheit der Patient*innen trat der SE nach Beginn der COVID-19-Symptome auf. Die meisten Patient*innen in dieser Arbeit hatten einen konvulsiven SE mit prominenten motorischen Symptomen. Die relativ geringe Repräsentation des nichtkonvulsiven SE (NCSE) in dieser Population und die Tatsache, dass in nur 70 % der in dieser Arbeit untersuchten SE-Patient*innen ein EEG durchgeführt wurde, gaben den Autoren Anlass zur Vermutung, dass NCSE unter anderem aufgrund von Limitationen in der EEG-Diagnostik bei infektiösen Patient*innen unterdiagnostiziert sein könnte [13, 19]. Eine epidemiologische Untersuchung aus Salzburg, Österreich, konnte keinen Unterschied in der Inzidenz des SE zwischen den Monaten der COVID-Pandemie März und April 2020, in denen in Österreich strikte Lockdown-Maßnahmen herrschten, und demselben Zeitraum in den Jahren 2018 und 2019 feststellen [28]. Es fand sich ein nichtsignifikanter Trend zu weniger Diagnosen von NCSE im Zeitraum der COVID-Pandemie in dieser Arbeit.

## Neuropädiatrische Aspekte bei COVID-19

Kinder aller Altersgruppen können von einer SARS-CoV-2-Infektion und COVID-19 betroffen sein [11]. Infektionen mit SARS-CoV‑2 sind bei Kindern wahrscheinlich genauso häufig wie bei Erwachsenen, allerdings entwickeln Kinder, besonders jene unter 10 Jahren, deutlich seltener COVID-19-Symptome oder einen schweren Krankheitsverlauf [49]. In einer retrospektiven Untersuchung von 1695 Patient*innen, die jünger als 21 Jahre waren und aufgrund einer schweren COVID-19-Erkrankung oder MIS‑C hospitalisiert waren, fanden sich bei 22 % neurologische Symptome [27]. Von diesen wiederum hatten 12 % eine schwerwiegende neurologische Manifestation. Unter anderem wurden schwere Enzephalopathien, Schlaganfälle, Enzephalitis und demyelinisierende ZNS-Erkrankungen sowie Fälle von Guillain-Barré-Syndrom beobachtet.

Fieberkrämpfe sind besonders mit viralen Infektionen wie Influenza und humanes Herpesvirus Typ 6, die hohes Fieber verursachen, assoziiert, aber treten auch in Zusammenhang mit Infektionen durch saisonale Coronaviren auf [9]. Eine Arbeit aus Rom, Italien, berichtete für den Zeitraum der COVID-19-Pandemie von März bis Mai 2020 eine signifikant höhere Zahl an Krankenhausaufnahmen aufgrund von Fieberkrämpfen im Vergleich zum Vorjahr [41]. Von 25 Kindern mit Fieberkrämpfen konnte allerdings nur bei 2 Kindern SARS-CoV‑2 mittels PCR aus dem Nasopharyngealabstrich nachgewiesen werden. Insgesamt sind die Hinweise für eine relevante Rolle von SARS-CoV‑2 für das Auftreten von epileptischen Anfällen (insbesondere Fieberkrämpfen) bei Kindern bislang gering.

## Behandlung von akut symptomatischen epileptischen Anfällen bei COVID-19

Eine erfolgreiche Behandlung von Patient*innen mit akut symptomatischen epileptischen Anfällen setzt voraus, dass diese als solche erkannt werden und die zugrunde liegende Ätiologie rasch identifiziert wird, um eine mögliche kausale Therapie frühzeitig etablieren zu können (z. B. mechanische Thrombektomie bei ischämischem Schlaganfall oder therapeutische Heparinisierung bei zerebraler Sinusvenenthrombose). Daneben werden Patient*innen mit akut symptomatischen Anfällen in der Regel vorübergehend mit Anfallsmedikamenten behandelt, um das Risiko für weitere Anfälle in der akuten Phase der zugrunde liegenden ZNS-Erkrankung zu reduzieren. In Zusammenhang mit COVID-19 wurde auf das mögliche Potenzial von pharmakokinetischen Interaktionen zwischen Anfallsmedikamenten und COVID-19-Therapien hingewiesen [16]. Beispielsweise können hepatische Enzyminduktoren wie Carbamazepin und Phenytoin die Konzentration von Remdesivir, das häufig in der Behandlung von schwer kranken COVID-19-Patient*innen eingesetzt wird, signifikant reduzieren [26]. Von der Universität Liverpool wird ein frei verfügbares Online-Tool zum Interaktionscheck von COVID-19-Therapien mit anderen Pharmaka angeboten [43].

## Epileptische Anfälle und COVID-19-Impfungen

Zum derzeitigen Stand (Mai 2021) sind 4 verschiedene Impfstoffe zur Prävention einer SARS-CoV-2-Infektion bzw. des Auftretens einer COVID-19-Erkrankung in der Europäischen Union zugelassen [15]. Die Internationale Liga gegen Epilepsie (ILAE) hat in einer Stellungnahme festgehalten, dass derzeit kein erhöhtes Risiko für das Auftreten von epileptischen Anfällen als Nebenwirkung von COVID-19-Impfungen bekannt ist [23]. Als Impfreaktion kann es zum Auftreten von Fieber kommen, was die „Krampfschwelle“ („seizure threshold“) herabsetzen kann. Im Zusammenhang mit Anfallsmedikation und COVID-19-Impfungen wurde darauf hingewiesen, dass bekannt ist, dass es nach Influenzaimpfungen zu durch Zytokine vermittelten Änderungen in der Expression von hepatischen Cytochrom-P450-Enzymen kommt und dadurch die Konzentration von Anfallsmedikamenten (z. B. Carbamazepin) beeinflusst werden kann [25]. Zu möglichen Interaktionen zwischen COVID-19-Impfungen und antiepileptischer Medikation liegen derzeit keine Informationen vor.

## Zusammenfassung

Es lässt sich zu den bisher vorliegenden Daten zu epileptischen Anfällen bei COVID-19 festhalten, dass epileptische Anfälle bei Patient*innen mit COVID-19 eine relativ seltene, aber wichtige neurologische Manifestation darstellen können. Ein Großteil der bisher berichteten Anfälle trat bei Patient*innen ohne Anamnese einer Epilepsieerkrankung auf. Für diese Anfälle dürfte ein akut symptomatischer Zusammenhang zur COVID-19-Erkrankung möglich bis wahrscheinlich zu sein. Bei einigen der berichteten Anfallsereignisse könnte es sich aber auch um unprovozierte epileptische Anfälle gehandelt haben, die nur in simpler Koinzidenz zur SARS-CoV-2-Infektion aufgetreten sind [44]. Zu beachten ist, dass es den derzeit vorliegenden Informationen zu Anfällen bei COVID-19 zum Teil an Details fehlt bzw. die zugrunde liegenden Arbeiten deutliche methodische Limitationen aufweisen. So wird in manchen Arbeiten nicht berichtet, ob Patient*innen mit Anfällen eine Anamnese von früheren Anfallsereignissen oder Epilepsie haben. In vielen Fällen fehlen Informationen zum diagnostischen Work-up, was die Beurteilung und ätiologische Zuordnung der berichteten Anfälle erschwert. Eine durch direkte Effekte des Virus auf das ZNS vermittelte Ätiologie der akut symptomatischen epileptischen Anfälle wie z. B. im Rahmen einer Enzephalitis scheint eher die Ausnahme zu sein. Das Zusammenspiel von multiplen Faktoren wie Hypoxie, Sepsis, Inflammation sowie schweren metabolischen Entgleisungen wie Hyponatriämie und Urämie, die sich bei COVID-19 häufig finden, könnte bei kritisch kranken Patient*innen das Auftreten von epileptischen Anfällen verursachen [20]. Zudem ist COVID-19, wie oben beschrieben, mit dem Auftreten mehrerer Erkrankungen des Gehirns assoziiert, für die wiederum selbst ein Zusammenhang mit akut symptomatischen epileptischen Anfällen bekannt ist. So treten akut symptomatische Anfälle bei 2–4 % aller Patient*innen mit ischämischem Schlaganfall und 10–18 % aller Patient*innen mit einer intrazerebralen Blutung auf [4]. Aus den bislang vorliegenden Informationen lässt sich vermuten, dass epileptische Anfälle eher bei Patient*innen auftreten, die schwerer von COVID-19 betroffen sind. Die Detektion von Anfällen kann bei kritisch kranken Patient*innen mit COVID-19, die invasiv beatmet werden und muskelrelaxiert sind oder eine Enzephalopathie/Bewusstseinsstörung aufweisen, unmöglich sein, sofern keine kontinuierliche EEG-Ableitung angewandt wird.

## Fazit für die Praxis

Epileptische Anfälle finden sich bei 1–2 % der Patient*innen mit COVID-19. COVID-19 kann zudem mit verschiedenen anderen neurologischen Komplikationen assoziiert sein, als deren Symptom epileptische Anfälle auftreten. Bei Patient*innen mit COVID-19 und epileptischen Anfällen sollte daher unbedingt weitere Diagnostik (z. B. zerebrale Bildgebung) erfolgen, um mögliche kausal behandelbare Ursachen rasch zu erkennen. Bei COVID-19-Patient*innen mit Bewusstseinsstörung und dem klinischen Verdacht auf das Vorliegen von epileptischen Anfällen sollte eine kontinuierliche EEG Ableitung durchgeführt werden. Vor der Anwendung von Anfallsmedikation sollte an die Möglichkeit von Interaktionen mit COVID-19-Therapien gedacht und ein Interaktionscheck durchgeführt werden.
